# Evaluation of LAMP for detection of *Shigella* from stool samples in children

**DOI:** 10.1099/acmi.0.000169

**Published:** 2020-09-14

**Authors:** Ramya Raghavan, Shouao Wang, Nandini Dendukuri, Sitanshu S. Kar, Subramanian Mahadevan, Barath Jagadisan, Jharna Mandal

**Affiliations:** ^1^​ Department of Microbiology, Jawaharlal Institute of Postgraduate Medical Education and Research, Puducherry, India; ^2^​ Centre for Outcomes Research, McGill University Health Centre – Research Institute, 5252 Boulevard de Maisonneuve W, Montreal PQ H4A 3S5, Canada; ^3^​ Department of Preventive and Social Medicine, Jawaharlal Institute of Postgraduate Medical Education and Research, Puducherry, India; ^4^​ Department of Pediatrics, Jawaharlal Institute of Postgraduate Medical Education and Research, Puducherry, India

**Keywords:** culture, diarrhoea, dysentery, LAMP, PCR, *Shigella*

## Abstract

**Background:**

To assess the diagnostic accuracy of loop-mediated isothermal amplification (LAMP) for the detection of *Shigella* from stool samples from children.

**Methods:**

Consecutive stool samples from children aged <13 years old who presented with acute watery diarrhoea or dysentery to the Department of Paediatrics were collected and processed in the Department of Microbiology. All the stool samples were subjected to culture, conventional PCR and LAMP. Genomic sequencing was performed for samples that were positive by LAMP but negative by both culture and conventional PCR. The LAMP results were compared to those from culture and to a composite reference standard based on culture and conventional PCR.

**Results:**

Amongst the 374 stool samples tested, 291 samples were positive by LAMP and 213 were positive by the composite reference standard. The sensitivity of LAMP was 100 % (98.3–100 %) and its specificity was 51.6 % (43.6–59.5 %) with a disease prevalence of 57 %. The sensitivity and specificity of LAMP improved to 99.3 % (94.2–100) and 98.2 % (94.5–99.9), respectively, using latent class analysis, while assuming that genomic sequencing has perfect specificity.

**Discussion:**

The authors have standardized the LAMP procedure for direct application to clinical stool samples. LAMP is a sensitive and specific method for the diagnosis of *Shigella* from stool samples of children as compared to both culture and conventional PCR.

## Background

*Shigella* is one of the leading causes of bacillary dysentery, the incidence of which is high in developing countries. Approximately 160 million cases of shigellosis with approximately 1.1 million deaths are reported annually [1]. Current estimates indicate that there is an increasing burden of shigellosis, warranting good control measures [2]. Unfortunately, the burden of shigellosis has probably been underestimated so far as it has relied on using stool culture, which has very low sensitivity. Hence there is a need to study the exact prevalence of shigellosis using methods that are more sensitive and specific. Detection of *Shigella* by targeting the *ipaH* gene by PCR has been found to be highly specific [3], indicating that it is a powerful molecular diagnostic technique for this purpose. Although PCR can detect culture-negative *Shigella *, technical difficulties limit its widespread use [3]. Loop-mediated isothermal amplification (LAMP), a rapid, reliable and cost-effective method, might be a good candidate to obviate such limitations. The LAMP technique has been validated for the identification of various pathogens and using various clinical samples. It has been applied for the detection of *Shigella* in food samples and artificially spiked stool samples [4]. In this study, we propose to apply the LAMP technique for the diagnosis of *Shigella* in stool samples from children and determine the sensitivity and specificity of the test.

## Methods

The study was conducted in the Department of Microbiology, Jawaharlal Institute of Postgraduate Medical Education and Research (JIPMER), a tertiary-care medical college hospital located in Puducherry, a town in southern India during the study period from July 2016 to December 2017. The study was approved by the Institute Ethics Committee (JIP/IEC/SC/2016/29/888). In preparing this manuscript we followed the STARD checklist for the reporting of diagnostic accuracy studies.

### Study population

Children who were less than 13 years of age who presented to the paediatric outpatient department or the emergency department with acute diarrhoea and dysentery were included in the study. The samples of neonates (less than or equal to 28 days) were excluded as *Shigella* is a rare pathogen in this age group. Stool samples of children with hospital-acquired diarrhoea (the onset of loose or watery stools at least 72 h after hospital admission), persistent diarrhoea and chronic diarrhoea were excluded. After informed consent and inclusion in the study, the stool samples of these children were collected and transported within 1 h to the Department of Microbiology’s laboratory.

The study involved stool samples from all eligible children with acute diarrhoea and dysentery. The data regarding symptoms and their duration were collected for every child and recorded in the data collection proforma.

For the purpose of the study, we used the following definitions.

- Acute diarrhoea was defined as any change in frequency and consistency, exemplified by passage of three or more loose stools per day or more frequently than normal for the individual, with a duration of <14 days.
- Dysentery was defined as any diarrhoeal episode in which loose or watery stools contained visible red blood and mucus.

### Sample size calculation

Based on the preceding year’s data on the number of *Shigella* isolates from stool cultures from children presenting to this institute with acute diarrhoea and dysentery, the proportion of culture positives was estimated to be 19.5 % (56 cultures positive for *Shigella* among the 286 stool samples of children with diarrhoea and dysentery tested). Using the proportion of 19.5 %, together with an expected sensitivity of 95 % and specificity of 95 % for culture, we determined that the sample size required to obtain a 95 % confidence interval for the true prevalence of *Shigella* with 5 % absolute precision was 374. Calculations were made using nMaster software version 2.0.

### Culture

All of the stool samples were assessed macroscopically and microscopically. In macroscopic stool examination, the stool was assessed for its consistency and for the presence of mucus and blood. The wet mount microscopy was examined at 10× and 40× magnification to look for pus cells, red blood cells, parasitic ova and cysts. All the samples were plated promptly upon reception at the laboratory. Due to logistical difficulties, we did not incorporate use of Cary–Blair medium. Some measures that would improve the culture isolation rates were implemented (stool samples were transported promptly to the laboratory for inoculation – within 1 h and if delay was anticipated the samples were quickly refrigerated). The stool sample was plated onto xylose lysine deoxycholate agar (XLD), deoxycholate citrate agar (DCA) and MacConkey agar. A loopful of stool sample was inoculated into selenite F broth. The samples were processed as per the standard protocol [5, 6]. The species were identified by using species-specific antiserum, which was obtained from Denka Seiken, Japan.

### Conventional PCR

The DNA from the stool samples were extracted using the QIAamp DNA stool minikit (Qiagen). Sixty microlitres of elute was obtained at the end of the extraction. The invasion plasmid antigen H (*ipa*H) gene was targeted to detect *Shigella *. The primers used and the conditions for DNA amplification were as described in the study by Thong *et al.* [7]. A 25 µl reaction volume consisting of 15 µl of master mix, 6 µl of sterile nuclease-free water, 1 µl each of forward and reverse primers of *ipaH* gene and 2 µl of template DNA was used. After amplification, 1.5 % agarose gel electrophoresis was carried out and *ipa*H gene was positive with a band size of 420 bp.

### LAMP

The primers used for LAMP were as described in a study by Sulong Li *et al*. [1]. The primers were of HPLC grade and they were reconstituted as per the standard protocol. Working concentrations of the primer stock were prepared as per Sulong Li *et al*. [1] and the LAMP assay was performed. The results were confirmed by gel electrophoresis. The final concentrations of BIP and FIP were estimated to be 10 µM each, and those of F3 and B3 were 50 µM each. Twenty-five microlitres of the reaction volume contained 15 µl of the master mix (Optigene LAMP kit, supplied by Ampligene, Ahmedabad, Gujarat, India.), 5 µl of DNA, 2.6 µl of primers consisting of 0.8 µl of 50 µM solutions of F3 and B3 each, 0.5 µl of 10 µM solutions of BIP and FIP each and 2.4 µl of nuclease-free water.

The LAMP was set at an amplification temperature of 63 °C for 1 h and the final termination step was at 80 °C for 5 min. All the LAMP amplicons were initially confirmed by gel electrophoresis. All species of *Shigella* were tested by LAMP, for which their DNA were extracted and subjected to LAMP using the above-mentioned standardized reaction conditions. As LAMP aids in the visualization of the amplicons by the naked eye, all the LAMP amplicons were visualized under the naked eye as well as ultraviolet rays for fluorescence (BioRad XR, USA). The LAMP products were also resolved by electrophoresis on a 1.5 % agarose Tris boric acid/EDTA gel with 1 µg ml^−1^ of ethidium bromide in an electrophoresis unit at 80 V for 45 min, and 5 µl of the LAMP product along with gel loading dye was visualized under an ultraviolet transilluminator and examined for a ladder-like band of amplification. Since the standardization was being performed for the first time, gel electrophoresis was performed to co-relate with the fluorescence detection. All the clinical samples were subjected to the above-mentioned standardized protocol.

### Threshold detection

To detect the threshold of the assay, varying concentrations of the *Shigella dysenteriae* type strain colonies ranging from 10^8^ to 10 colony-forming units (c.f.u.) ml^−1^ were prepared and subjected to LAMP assay. The type strain used was a laboratory-isolated and sequenced strain. The GenBank accession numbers for the virulence genes of the type strain are as follows: *set1A* gene, KR822808; *ipaH* gene, KR269602; *ial* gene, KT013267. To look for any change in threshold for detection by possible inhibitors, autoclaved stool sample were mixed with varying concentrations of *S. dysenteriae* type strain colonies. DNA extracted from these samples were subjected to LAMP. Although use of reference strains would have been ideal, we did not have the ATCC/NCTC reference strains at our laboratory. Hence, we opted for a genetically sequenced type strain.

### Specificity of LAMP

**Fig. 1.**
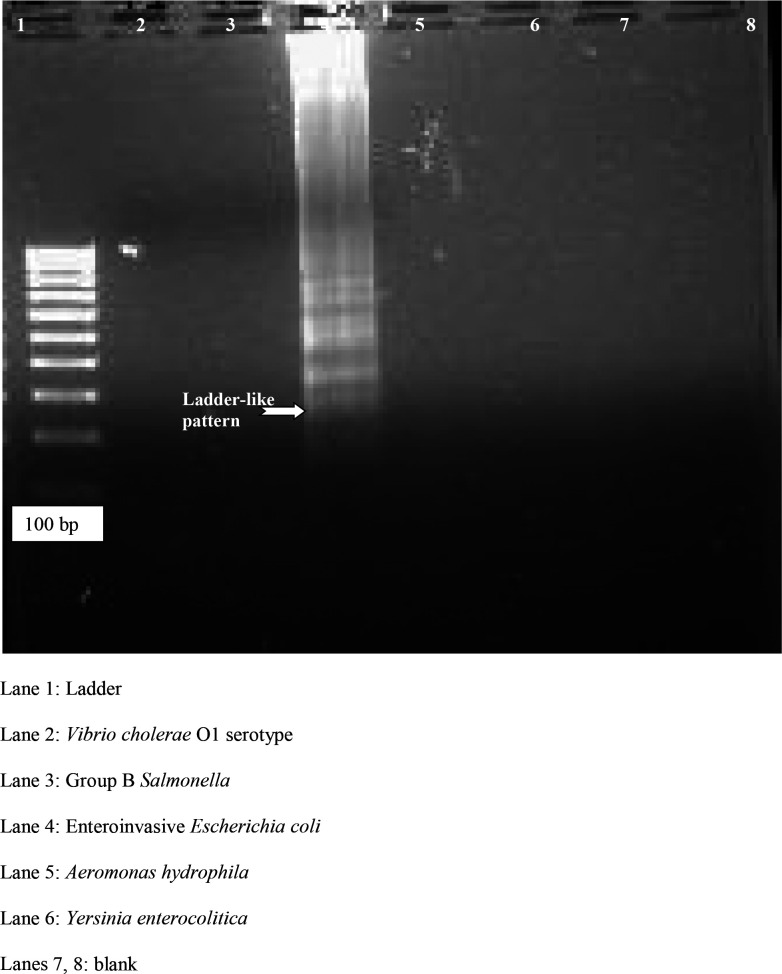
Specificity of LAMP assay. Lane 1, ladder; lane 2, *V. cholera* O1 serotype; lane 3, group B *Salmonella *; lane 4, enteroinvasive *E. coli *; lane 5, *A.s hydrophila*; lane 6, *Y. enterocolitica *; lanes 7 and 8, blank.

To determine the specificity of LAMP, extracted DNA (boiling method) [8] of different pathogens such as *Yersinia enterocolitica *, *Vibrio cholerae *, enteroinvasive *Escherichia coli* (EIEC), group B *Salmonella *, *S. dysenteriae *, *Shigella flexneri *, *Shigella boydii *, *Shigella sonnei* and *Aeromonas hydrophila* were subjected to the LAMP using the standardized reaction conditions mentioned above to look for any cross-reactions. There were no cross-reactions. All species of *Shigella* and EIEC gave positive LAMP amplification (Fig. 1). A LAMP assay was also conducted for healthy children’s stool samples to look for any non-specific amplification.

### Genomic sequencing

The LAMP amplicons from the samples that were negative by both culture and conventional PCR were amplified by the conventional PCR method using F3 and B3 primers. They were resolved by using 1.5 % agarose gel electrophoresis to look for a band with a size of 210 bp. These PCR-amplified products were subjected to sequencing using an ABI 3700 sequencer (Applied Biosystem, Foster, CA, USA) at BioServe Biotechnologies (India) private Ltd, India. Contig sequences generated were assembled and compared using the Basic Local Alignment Search Tool (blast) of the National Center for Biotechnology Information (NCBI).

### Statistical analysis

We compared LAMP to culture (the gold standard) or to a composite reference standard (defined as *Shigella *-positive=culture-positive or conventional PCR-positive). In both cases, the reference standard was assumed to be perfect. However, these conventional methods are known to be biased as both culture and conventional PCR have sub-optimal sensitivity, although they have high specificity. Therefore, in addition to the traditional analysis, we carried out a latent class analysis that allows us to acknowledge that all three available tests – culture, conventional PCR and LAMP – are imperfect. We carried out two separate analyses, one based on the test results alone and one also considering genetic sequencing results for the *ipaH* gene, which was carried out on a subset of subjects.

In applying the latent class analyses we made several assumptions:

- the specificity of culture ranges from 0.99 to 0.999;
- the specificity of conventional PCR ranges from 0.975 to 0.985;
- there is a possibility of correlated errors between the two PCR tests, i.e. they can both be false positive or false negative simultaneously;
- genetic sequencing has perfect (100 %) sensitivity.

We also made two different assumptions regarding the specificity of genetic sequencing. In the first analysis, we considered it to be unknown. In the second analysis, we assumed that it ranges from 0.975 to 0.985, reflecting the fact that the sample at hand only included symptomatic patients with acute diarrhoea, thereby limiting the risk of false positives that may arise due to the presence of non-viable DNA from a recent infection.

The conventional analysis was carried out in SPSS software (version 22) using methods for the estimation of a proportion. The latent class model was estimated using a Bayesian approach via the rjags library in the R statistical software package (the model we used is listed in the annexure) [9]. We used non-informative beta (1, 1) prior distributions over the disease prevalence, the sensitivity and specificity of LAMP and the sensitivities of culture and conventional PCR.

## Results

**Fig. 2.**
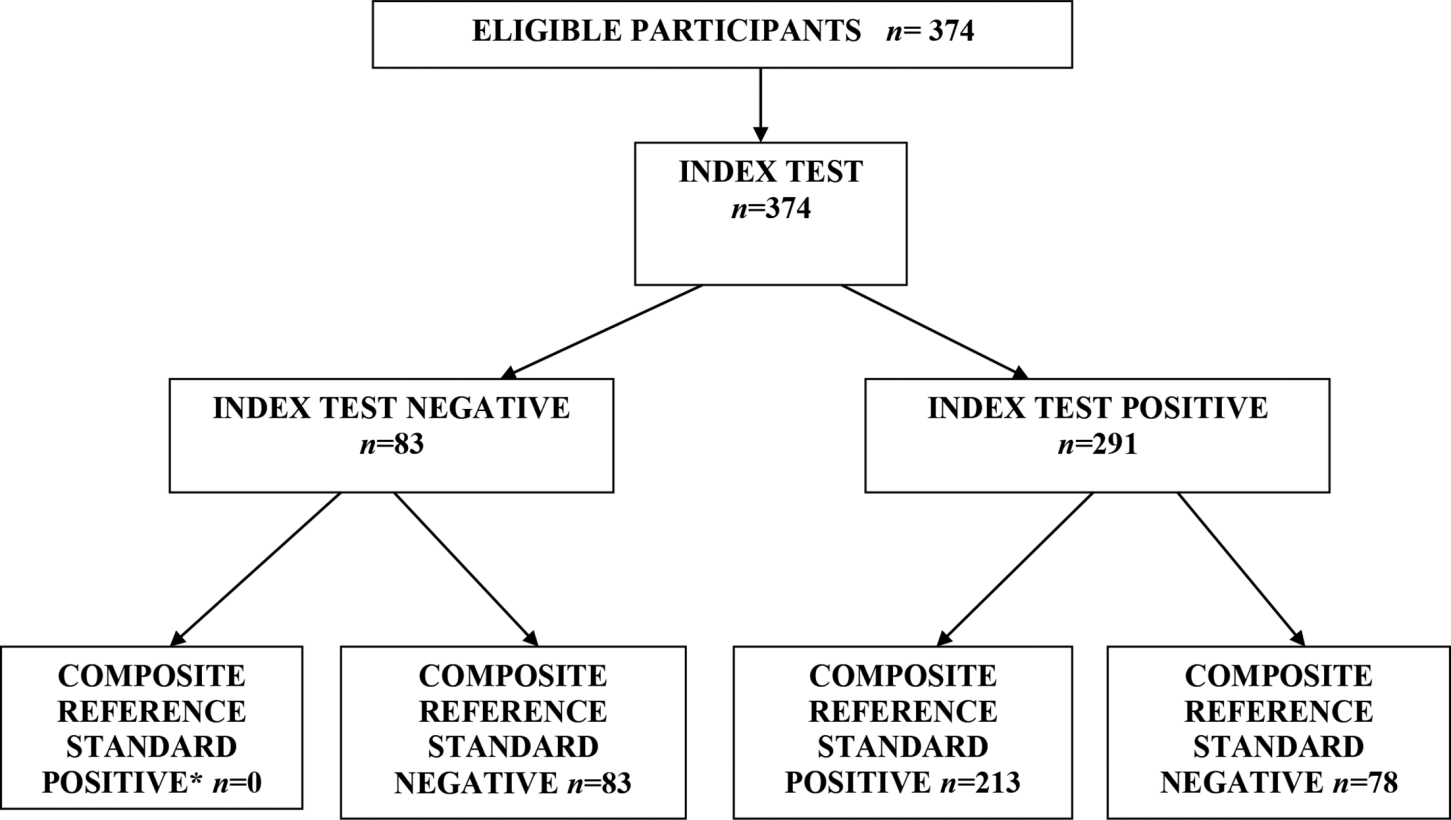
Flowchart summarizing recruitment of subjects in the study. *Composite reference standard positive, culture- or conventional PCR-positive.

A flow diagram summarizing the recruitment of children in the study is presented in Fig. 2. Of the 374 children included in the study, 159 were girls and 215 were boys. The median age of the study population was 15±3.85 months. The average duration of symptoms was 3.6±1.7 days. Seventy-eight children (21 %) presented with blood in stools, whereas 296 (79 %) presented with acute watery diarrhoea and 26 out of 374 (7 %) samples tested positive for *Shigella* by culture (Table 1). Amongst the *Shigella* spp. isolated, *S. flexneri* and *S. sonnei* were the most commonly isolated species; both were isolated from 13 (50 %) samples. *S. dysenteriae* and *S. boydii* were not isolated from the study population.

**Table 1.** Distribution of results of tests and genetic sequencing

Conventional PCR	LAMP	Culture	Genetic sequencing of *ipaH* gene	Frequency
+	+	+	Not done	23
+	+	−	Not done	187
−	+	+	Not done	3
−	+	−	Done and +	78
−	+	−	Done but −	0
+	−	+	Not done	0
+	−	−	Not done	0
−	−	+	Not done	0
−	−	−	Not done	83

### Detection limit of conventional PCR

**Fig. 3.**
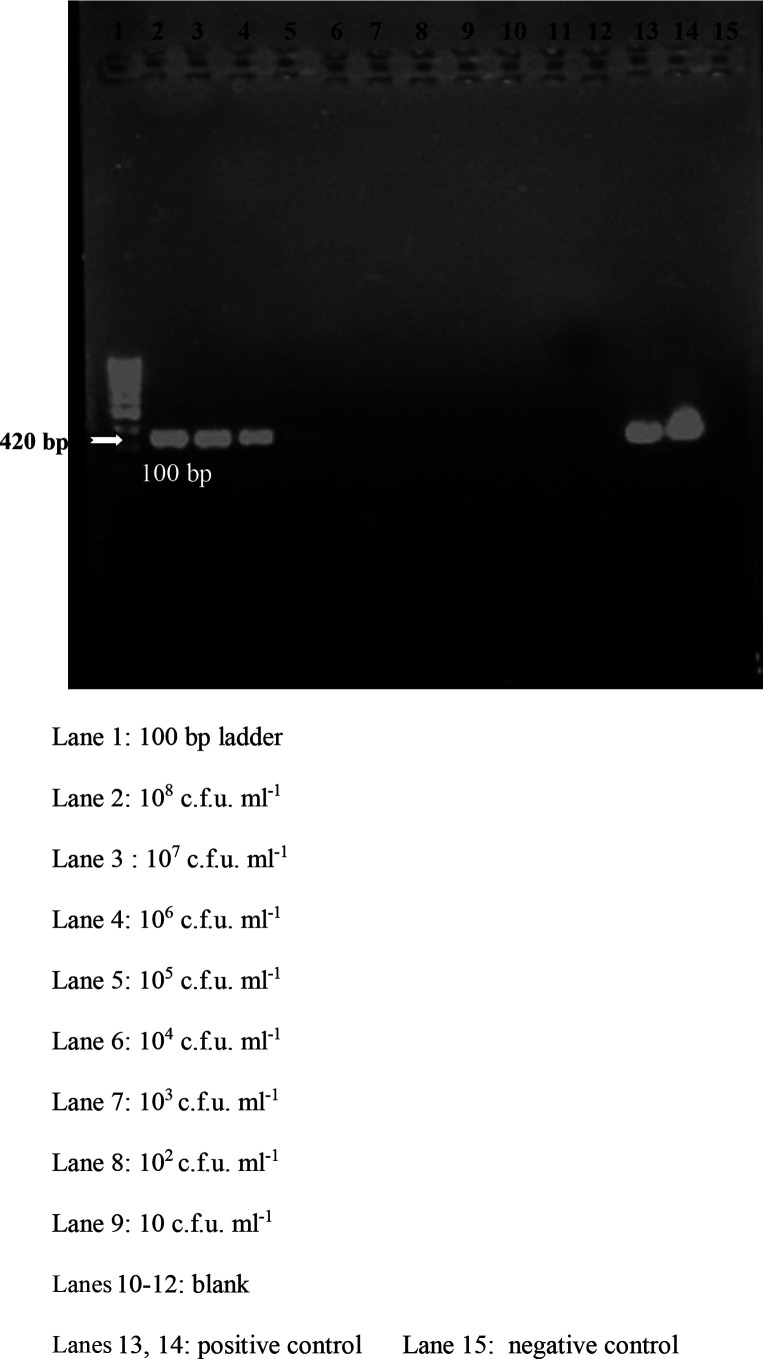
Threshold of conventional PCR. Lane 1 :, 100 bp ladder; lane 2, 10^8^ c.f.u. ml^−1^; lane 3, 10^7^ c.f.u. ml^−1^; lane 4, 10^6^ c.f.u. ml^−1^; lane 5, 10^5^ c.f.u. ml^−1^; lane 6, 10^4^ c.f.u. ml^−1^; lane 7, 10^3^ c.f.u. ml^−1^; lane 8, 10^2^ c.f.u. ml^−1^; lane 9, 10 c.f.u. ml^−1^; lanes 10–12, blank; lanes 13 and 14, positive control; lane 15, negative control.

The detection threshold by spiking the liquid broth was found to be 10^5^ c.f.u. ml^−1^. (Fig. 3)

### Detection limit of LAMP

**Fig. 4.**
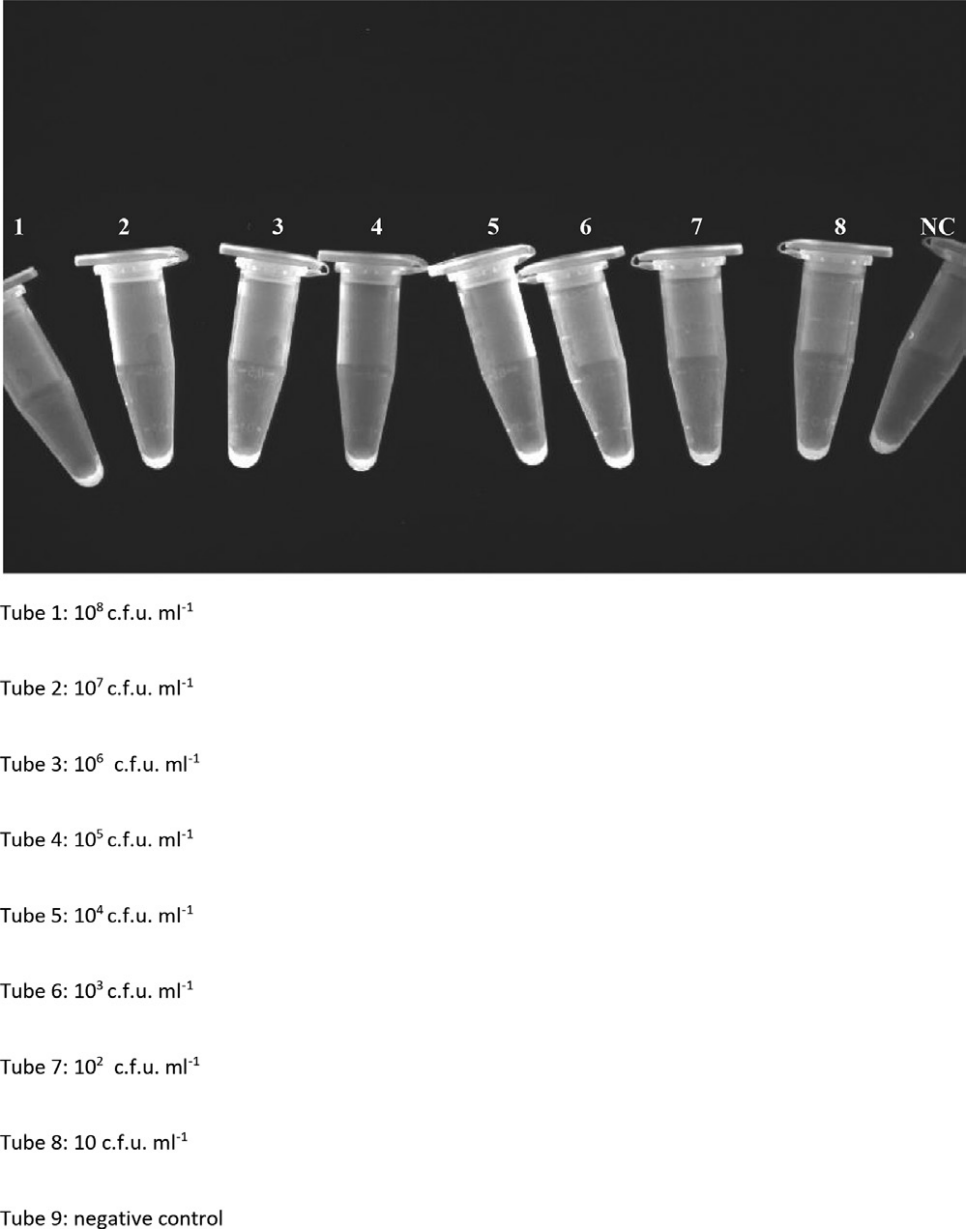
Threshold of LAMP. Tube 1, 10^8^ c.f.u. ml^−1^; tube 2, 10^7^ c.f.u. ml^−1^; tube 3, 10^6^ c.f.u. ml^−1^; tube 4, 10^5^ c.f.u. ml^−1^; tube 5, 10^4^ c.f.u. ml^−1^; tube 6,10^3^ c.f.u. ml^−1^; tube 7, 10^2^ c.f.u. ml^−1^; tube 8, 10 c.f.u. ml^−1^; tube 9, negative control.

LAMP was able to detect *Shigella* down to a concentration of 10 c.f.u. ml^−1^. (Fig. 4) Thus, the detection limit is 10 c.f.u. ml^−1^ and this concentration is required in a sample for the LAMP to be positive.

There was no cross-reactivity when using healthy children’s stool samples. All the LAMP products that were amplified showed increased fluorescence and no fluorescence was noted in the negative control. The distributions of the three tests are presented in Table 1. A total of 210 samples were positive for the *ipa*H gene (56.2 %) by conventional PCR as compared to 291 (77.8 %) by LAMP (Table 2). Estimates of the prevalence of *Shigella* based on the two conventional methods are given in Table 2. As expected, the composite reference standard resulted in a much higher prevalence. The sensitivity of LAMP was perfect when compared to culture as all samples detected by culture were also detected by LAMP. However, the specificity estimated for LAMP compared to culture was very poor at 25 %. This was possibly because negative culture results incorrectly classified some true *Shigella *-positive cases as disease-negative. This problem was rectified to an extent by using a composite reference standard, but the specificity remained very low at 51 %. Therefore, we also performed a latent class analysis.

**Table 2.** Estimates (and 95 % uncertainty intervals) of *Shigella* prevalence, and the sensitivity and specificity of the tests based on the different statistical analyses

Parameter	Culture as a reference standard	Composite of culture and conventional PCR	LCA of the three tests alone	LCA of three tests and genetic sequencing	
				Assuming unknown specificity of genetic sequencing	Assuming perfect specificity of genetic sequencing
Prevalence of *Shigella *	6.95 (4.6–10)	57 (51.8–62)	65.5 (56.1–77.8)	70.6 (57.4–82.6)	78.2 (73.4–83.1)
Sensitivity of LAMP	100 (86.8–100)	100 (98.3–100)	99.1 (89.7–100)	99.3 (92.6–100)	99.3 (94.2–100)
Specificity of LAMP	25 (20.5–29.9)	51.6 (43.6–59.5)	62 (47.3–91.6)	73.2 (50.3–99.2)	98.2 (94.5–99.9)
Sensitivity of culture	–	–	10.7 (7.1–15.3)	10.1 (6.7–14.8)	9.0 (6.1–12.6)
Specificity of culture	–	–	99.6 (99.1–99.9)	99.6 (99.1–99.9)	99.6 (99.1–99.9)
Sensitivity of conventional PCR	88.5 (69.9–97.6)	–	84.9 (71.4–95.7)	78.9 (66.5–94.5)	71.3 (65.6–76.4)
Specificity of conventional PCR	46.3 (40.9–51.7)	–	98.1 (97.5–98.5)	98.1 (97.5–98.5)	98.1 (97.5–98.5)

Based on the latent class analysis of the three tests alone, the prevalence of *Shigella* was 65.5 % (56.1–77.8 %), the sensitivity of LAMP was 99.1 % and the specificity of LAMP was 62 %. Thus, compared to the conventional analyses, there was little change in the sensitivity estimate but the specificity estimate was increased. However, there remained considerable uncertainty around this estimate. Although the upper bound of the specificity exceeded 90 %, the median value of 62 % was lower than the desired standard for a diagnostic test. The latent class analysis also provided estimates of the accuracy of the two conventional tests in addition to that of LAMP. We can see that the sensitivity of culture is very low 10.7 %, while the sensitivity of conventional PCR is much higher at 85 %.

When we incorporated the partial information available on gene sequencing into the analysis, we found that the specificity of LAMP increased further. If we assume that gene sequencing has perfect specificity, then this would suggest that the specificity of LAMP is very high and comparable to that of conventional PCR. This would suggest that LAMP is suitable for use as a diagnostic test in comparable populations where the duration of symptoms is acute. Other parameters (e.g. the sensitivity of the three tests) did not change appreciably when gene sequencing results were added.

## Discussion

The study was performed with the objective of assessing the diagnostic accuracy of LAMP for the detection of *Shigella* spp from stool samples of children as compared to a composite reference standard (culture and conventional PCR). The study was performed in a tertiary care hospital in southern India. The overall estimated disease burden among the paediatric population based on LAMP was found to be 57 %. The estimated proportion of *Shigella* using LAMP technique was high as compared to the estimated proportion based on stool culture. This is in accordance with several previous studies, where the proportion was reported to be high when sensitive molecular methods were applied [10, 11]. The detection threshold for stool culture to isolate the organism was found to be 10^7^ bacilli ml^−1^ stool. With a detection limit of 10^7^, culture remained an inefficient test to detect *Shigella* spp. LAMP and conventional PCR, which were found to have a detection threshold of 10 copies ml^−1^ and 10^5^ copies ml^−1^, respectively, understandably had higher sensitivities and reflected the true disease burden. Liu *et al*., in a recent study, showed that the burden of many organisms was underestimated when conventional methods were used for detection. They found that the burden of *Shigella* spp. or EIEC was higher than expected when quantitative PCR was used for detection of the organism [12].

The most common clinical presentation amongst the study population was found to be acute watery diarrhoea (79 %) followed by fever (47 %). Dysentery was present in 21 % of the children in the study. This was similar to a study conducted by Kosek *et al*., where the major clinical presentation was found to be acute watery diarrhoea [13]. It is known that many cases of shigellosis present with secretory diarrhoea in the initial phase of illness [14]. For reporting cases and outbreaks of shigellosis, the World Health Organization (WHO) suggests that the standard case definition of bloody diarrhoea or dysentery, i.e. diarrhoea with visible blood in the stool, is used [5]. The notion that shigellosis most often presents as dysentery is long held. With the advent of molecular diagnostics, this has been challenged, and it has been shown that in the initial phases of the disease the manifestations probably mimic any watery diarrhoea [15].

Conventional PCR was positive in 56 % of the study subjects, which was higher than for culture. This is in line with the literature, where it has often been found that the sensitivity of molecular techniques are high compared to culture methods [16, 17]. An increase in sensitivity would mean an increase in the estimated prevalence. In a study performed by Vu *et al*., it was found that relying on culture resulted in an underestimation of the burden of shigellosis. Applying a PCR technique, the authors were able to demonstrate a 37 % increase in the burden of shigellosis [18]. This was also true in the present study; the estimated burden using the composite reference standard of culture and PCR was 57 % as compared to the prevalence of 7 % obtained using only culture. Since the PCR target was genus-specific, species identification could not be done. The *ipaH* gene is also found in EIEC. In this study, EIEC and *Shigella* could not be differentiated. However, previous studies have shown that the incidence of EIEC is very low in these regions. Hence, the *ipa*H gene detected is more likely to be due to the presence of *Shigella* in the sample. In regions where EIEC is known to have a very low prevalence, *ipaH* positivity in the stool is more likely to be due to *Shigella *. Thiem *et al*. used a similar assumption in their study [17]. We based our thinking on a careful review of these studies. The findings are no different even in studies performed in various parts of India. In studies performed on diarrhoegenic *E. coli *, the prevalence of EIEC is low. In a study performed by Taru Singh *et al*., there were no isolates of EIEC among the study population [19], whereas in a study performed by Hegde *et al*. the prevalence was found to be 1.5 % [20]. In a study performed by Rajendran *et al*., EIEC was found to have a prevalence of 1 % amongst the DEC isolates [21]. All these studies were performed in various regions of India. Comprehensive phylogenetic studies of *Shigella* and *E. coli* have proven the genetic diversity of the EIEC and so the use of *uidA* and *lacY* to differentiate the two organisms was not as accurate as previously thought [22,23]. Given the cost and the logistics involved, we decided not to include the additional step of multiplex PCR for *uidA* and *lacY* genes.

LAMP was found to have a sensitivity of 100 % [95 % confidence interval (CI)=98.3–100 %] and a specificity as 51.6 % (95 % CI=43.6–59.5 %) as compared to the composite reference standard of culture and conventional PCR. Seventy-eight samples that were negative by the composite reference standard tested positive by LAMP. The threshold for detection for conventional PCR was found to be 10^5^ copies ml^−1^, whereas it was only 10 copies ml^−1^ for the LAMP. Thus, LAMP was more sensitive than conventional PCR and may reflect the true burden of shigellosis if applied in epidemiological studies. This finding of ours is in line with the existing literature. In a study performed by Song *et al*., where artificially spiked stool samples were subjected to both PCR and LAMP, it was shown that PCR failed to detect *Shigella* at concentrations <6.5×10^7^ c.f.u. ml^−1^, whereas LAMP could [4].

It is important to note that a highly sensitive technique in the absence of an improper gold standard shows a low specificity [24–26]. This holds true for this study too. When compared to the imperfect gold standard, i.e stool culture alone, LAMP had a very low specificity of 25 % (95 % CI=20.5–29.9). But, on comparison with the composite reference standard, the specificity of the test was found to be 51.6 % (95 % CI=43.6–59.5). All those samples that were negative on the composite reference standard but were positive on LAMP were subjected to meta-genomic sequencing. The sequencing of these amplicons was positive for the *ipaH* gene in all these samples, proving that they were indeed true positives.

Three of the samples tested for shigellosis were positive by culture but negative by conventional PCR. LAMP was positive for all three of these samples (Table 1). This could be due to the fact that conventional PCR can be affected by stool inhibitors but LAMP is not [27]. In many diagnostic accuracy studies, an imperfect gold standard is cited as a limitation in the evaluation of a newer diagnostic technique. [28–30]. Some recent work has shown that the composite reference standard approach can give rise to considerable bias as it makes assumptions that are known to be wrong, i.e. that the composite reference standard is a perfect test [31]. Bayesian latent class analysis (BLCA) would therefore seem to be the preferred approach, as it avoids this assumption.

The prior distributions in BLCA can be estimated based on a review of the literature and/or expert opinion in the absence of data [29]. The existing literature does not provide estimates of culture specificity for shigellosis and the specificity of conventional PCR is only estimated with respect to culture, which is known to be imperfect. Therefore, for the Bayesian analysis, we used subjective expert opinion to provide a range of plausible values for the specificities of these two tests. The authors used informative prior distributions only for those parameters that are well established in the literature. For the culture and PCR tests we made no assumptions regarding the sensitivities. The authors only assumed that the specificities were very high but not perfect, in keeping with what is well documented in the published literature. In the case of genetic sequencing, we assumed that the sensitivity was 100 %, but we present two separate analyses, one where no assumption was made about the specificity and the other where the specificity was assumed to be high but not perfect.

We found that the sensitivity of LAMP obtained by comparison with culture as a gold standard was very similar to the estimates obtained by comparison with a composite reference standard of culture and conventional PCR as well as by BLCA (Table 2). Interestingly, there was a remarkable difference in the specificity of the test between the methods. The specificity of LAMP was higher by latent class analysis that adjusted for the imperfect sensitivity of culture. We found that the assumptions regarding the specificity of genetic sequencing had an important influence on the specificity of LAMP; it ranged from 73.2 (when the genetic sequencing was assumed to be of unknown specificity) to 98.3 % (when the genetic sequencing was assumed to have perfect specificity) (Table 2). We therefore believe that the BLCA approach reflects the true test performance. Similar observations have been made by other researchers where they have found that application of BLCA resulted in better estimation of the diagnostic test than the traditional way of analysis when the gold standard test was imperfect [29, 32, 33].

The strengths of our study may be summarized as follows.

- The LAMP procedure was well standardized before testing on study samples. This standardized protocol was then followed for all the study samples.
- LAMP-positive samples could be easily differentiated from negative samples simply by the turbidity of the reaction mixture. All the LAMP reactions were read by two independent investigators, who were blinded to each other’s interpretation, thereby reducing subjective errors.
- Sequencing of LAMP products was performed to ensure that there were no false positives.
- To address the issue of an imperfect gold standard, comparison with a composite reference standard as well as BLCA were performed. This is the first time BLCA has been performed to compare the performance of various diagnostic tests for *Shigella *.

The present study is not without limitations. The following are some of the limitations of the study.

- Data regarding antibiotic use prior to presentation were a part of the study proforma. However, the details were often incomplete. Many of the samples were obtained from patients attending the outpatient department, making it difficult to obtain the information later on. As per guidelines, children with watery diarrhoea do not need antibiotics. So, we assume most of the children in the study would not have received antibiotics recently. But a few children could have received empirical antibiotics, albeit irrationally. Children with dysentery could have been given antibiotics. In such a situation, where the child has received one or two doses of antibiotics before the stool specimen is taken for tests, stool culture is likely to be negative, as *Shigella* is an extremely delicate organism. Although there was a true infection, one or two doses antibiotics could have resulted in negative results on culture. However, conventional PCR or LAMP or gene sequencing would still be positive, as they can detect even small amounts of DNA. A sub-set analysis would have been helpful to evaluate the use of molecular methods in the antibiotic-exposed children among the study population.
- Both PCR and LAMP only identified the presence of the *ipaH* gene. We did not differentiate between EIEC and *Shigella* since currently there are no foolproof techniques to differentiate them. Previous studies have shown that the incidence of EIEC in Asian regions is exceedingly low [34]. Hence, we assumed that the sources of all *ipaH*-positive samples were *Shigella *. Further, speciation could not be performed.
- Comparison with real-time PCR rather than conventional PCR would have been ideal, as the former is more sensitive. However, we could not do this due to logistical and financial restrictions.

## Conclusion

LAMP is a sensitive and specific method for the diagnosis of *Shigella* from stool samples of children as compared to both culture and conventional PCR. We have standardized the LAMP procedure for direct application to extracted stool samples. The estimated prevalence of *Shigella* is higher than expected. The prevalence of *Shigella* among children presenting with only acute watery diarrhoea is high. Thus, using only the definition of dysentery for surveillance may result in missing a significant proportion of cases. The WHO case definition of shigellosis must be revisited in the light of these observations. Community-based surveillance studies are required. For surveillance studies, molecular tools such as LAMP must be used, as they will help in estimating the prevalence of the disease. Such epidemiological studies are required to determine the true burden of the disease. Longitudinal follow-up studies to determine how long LAMP remains positive in a child affected by shigellosis are a must. Such studies would be useful to study the performance of the technique in patients who have been treated with antibiotics.
